# Uterine Histone Secretion Likely Fosters Early Embryo Development So Efforts to Mitigate Histone Cytotoxicity Should Be Cautious

**DOI:** 10.3389/fcell.2017.00100

**Published:** 2017-11-27

**Authors:** Lon J. Van Winkle

**Affiliations:** ^1^Department of Biochemistry, Midwestern University, Downers Grove, IL, United States; ^2^Department of Medical Humanities, Rocky Vista University, Parker, CO, United States

**Keywords:** histones, blastocyst, implantation, antimicrobial, early development, amino acid transport systems

## Introduction

The possible benefits of mitigating extracellular histone cytotoxicity have been outlined for the reproductive tract and other organs (e.g., Simon et al., 2013; Galuska et al., 2017; Lefrançais and Looney, 2017; Wygrecka et al., 2017; Yang et al., 2017). However, a reassessment of previously published data supports the notion that uterine histone secretion fosters early embryo development in multiple ways. (See below.) Hence, efforts to neutralize extracellular histone action in reproductive organs should be cautious. Thus far, there appears to be little discussion of how to preserve desirable histone effects while mitigating pathology caused by excessive extracellular histone actions.

## Mechanisms of histone cytotoxicity are likely related to their antimicrobial effects

Histones contribute to eukaryotic chromatin structure and function in a well-known manner (e.g., Harr et al., 2016). Less well known are the extra-nuclear functions of these macromolecules (e.g., Parseghian and Luhrs, 2006). Among these other functions, extracellular histones fight bacterial, fungal, viral, and other parasitic infections (Papayannopoulos, in press). At least two distinct mechanisms account for these antimicrobial effects.

First, histones are essential components of neutrophil extracellular traps (NETs). NETs are net-like structures that form from decondensed chromatin and cytosolic proteins. They usually form when neutrophils undergo cell death via a process termed NETosis. NETs trap and kill each of the pathogens listed above (Papayannopoulos, in press).

Free histones also have antimicrobial functions (Kawasaki and Iwamuro, 2008). For example, histones in amniotic fluid appear to fight bacteria by neutralizing the lipopolysaccharide (LPS) of microbes that gain access to this fluid (Witkin et al., 2011). Without such protection, LPS could cause pre-term labor and delivery (Hirsch et al., 2006).

The cytotoxic effects of histones are likely related to their mechanisms of antimicrobial action. For example, the hyperinflammatory response and death of mice given high doses of LPS appear to require free histones. Moreover, administration of free histones to mice causes their death (Xu et al., 2009). Similarly, histones contribute to organ damage by excessive NETosis especially in lungs (Silk et al., 2017; Papayannopoulos, in press). Clearly, NETosis and the actions of free histones need tight regulation in order to benefit mammals and prevent pathological effects.

## Antimicrobial actions of histones in the reproductive tract

Extracellular histones also help to inhibit microbial proliferation in the reproductive tract. For example, in a mouse model, NETs appear to limit Group B Streptococcal infection via the vagina during pregnancy (Kothary et al., 2017). In addition, free histones in fluids from the reproductive tract of cows exhibit antimicrobial actions (Dráb et al., 2014). Since pathogens can cause inflammation in the reproductive tract of mammals, they can also adversely affect mammalian reproduction (Wiesenfeld et al., 2002; Mårdh, 2004; BonDurant, 2007). While extracellular histones protect against microbes in at least two ways, only one of these mechanisms of action likely apply to free histone molecules in follicular, oviductal, and uterine secretions.

## Direct contribution of histones to early embryo development

### Background

Histones appear most abundantly in human uterine secretions at the time the uterus is receptive to blastocyst implantation (Beier and Beier-Hellwig, 1998). Similarly, histones are synthesized at increased rates in the uterine epithelium and stroma of ovariectomized mice upon administration of a hormonal protocol known to result in blastocyst implantation about 25 h later (Smith et al., 1970). Assuming histones appear in mouse uterine fluid when the uterus becomes receptive to blastocyst implantation, what other functions might histones serve there? One good possibility involves amino acid transport system B^0, +^ in mouse and probably human blastocysts (Van Winkle et al., 2006). In order to consider this possibility in context, we first review system B^0, +^ involvement in early embryo development and blastocyst implantation in the uterus.

The process of blastocyst implantation in the mouse is especially amenable to study owing to experimentally-controlled delay of implantation. While delay of implantation (diapause) occurs naturally in mice when blastocysts develop in nursing mothers, it can be produced experimentally in mice by removing their ovaries about 76 h after their eggs have been fertilized (Van Winkle and Campione, 1987; Van Winkle et al., 2006). Daily administration of progesterone followed by estrogen on day 7 of pregnancy then leads to blastocyst implantation 25 h later.

During this activation from delay of implantation, signaling owing to leucine uptake via amino acid transport system B^0, +^ results specifically in development of trophoblast motility and penetration of the uterine epithelium by blastocysts (Van Winkle et al., 2006). This signaling occurs because increases in the Na^+^ and K^+^ concentrations in uterine secretions about 6 h after estrogen administration to ovariectomized, progesterone-treated rodents (Van Winkle et al., 1983; Nilsson and Ljung, 1985) drive net Na^+^-dependent system B^0, +^ leucine uptake by the blastocyst trophectoderm. Leucine then stimulates the mTOR signaling that is needed for development of trophoblast motility and penetration of the uterine epithelium, which occur about 19 h later (Van Winkle et al., 2006). Meanwhile the uterine environment somehow suppresses system B^0, +^ activity beginning about 10 h after estrogen administration (Lindqvist et al., 1978; Van Winkle and Campione, 1987). For example, blastocysts take up a radiolabeled, nonmetabolizable amino acid *in utero*, when it is administered to their mothers about 6 h after estrogen administration, but little or no uptake occurs when the amino acid is administered 4 h *before* or *after* this time (Lindqvist et al., 1978). We calculated that the decrease, in the rate of amino acid uptake between about 6 and 10 h after estrogen administration, is statistically equivalent to finding a drug that lowers the death rate from 94 to 6% (based on the effect size that can be calculated from the data of Lindqvist et al., 1978). At the time of the latter report, however, the full physiological significance of these changes in amino acid uptake by blastocysts *in utero* were not understood.

Although system B^0, +^ is relatively inactive *in utero* during the 15 h prior to blastocyst implantation, it can be reactivated to even greater levels of transport activity simply by removing blastocysts from the uterus near the time of blastocyst implantation (Van Winkle and Campione, 1987). This ability to reactivate system B^0, +^ activity also likely serves an important physiological function (Van Winkle et al., 2006). After reactivation, system B^0, +^ would help to remove tryptophan from the implantation chamber during the initial penetration of the uterus by motile trophoblasts and, thus, help to suppress T-cell proliferation and immunologic rejection of the blastocyst (Munn et al., 1998; Baban et al., 2004).

### Possible histone involvement

But what reversibly suppresses system B^0, +^ activity beginning about 15 h before blastocyst implantation? Good candidates include histones that are likely secreted by uterine epithelial and possibly stromal cells when the uterus becomes receptive to blastocyst attachment and penetration (Smith et al., 1970). At near the histone concentrations detected in uterine fluid (Beier and Beier-Hellwig, 1998; Dráb et al., 2014), we found these macromolecules to inhibit amino acid uptake by mouse blastocysts. System B^0, +^ activity, in particular, was inhibited much more than the activities of several other amino acid transport systems in blastocysts (Table IV in Van Winkle, 1993). In fact, the extent to which histones inhibited each of four different amino acid transport systems in blasocysts, differed from each other (*p* < 0.02), and ranged from near 90% inhibition of system B^0, +^ to no inhibition of system L. Hence, it seems unlikely that histone cytotoxicity alone caused histone inhibition of amino acid transport system B^0, +^ activity in blastocysts.

Perhaps not coincidentally, the effect size of this system B^0, +^ inhibition by histones equals the effect size reported above, for reduction of the rate of amino acid transport into blastocysts *in utero* between about 6 and 10 h after estrogen administration to progesterone-maintained ovariectomized mice (Lindqvist et al., 1978). In addition, histone H2A (one of the more conspicuous histones in secretions of the receptive human uterus; Beier and Beier-Hellwig, 1998) likely is more effective at inhibiting amino acid uptake by blastocysts than other histones (Doman and Van Winkle, 1979). Reactivation of system B^0, +^ in blastocysts, at the time of blastocyst penetration of the uterine epithelium, could be accomplished simply by removing histones from the relatively small amount of uterine fluid in implantation chambers. In this regard, proteases, needed to hydrolyze histones to inactive products, appear to abound in these chambers (e.g., Afonso et al., 1997).

While it is unclear why system B^0, +^ activity needs to be suppressed after mTOR signaling, we observed one tantalizing possibility. When we incubated delayed-implantation blastocysts for 25 h *in vitro* in medium containing a relatively high Na^+^ concentration, they irreversibly lost their Na^+^-dependent component of amino acid uptake (Van Winkle, 1977). This apparent loss of Na^+^-dependent system B^0, +^ activity would likely mean that the ability of blastocysts to activate net Na^+^-dependent tryptophan uptake would also be lost. If such loss were to occur in the implantation chamber *in utero*, then implanting blastocysts could face immunological rejection. Hence, suppression of system B^0, +^ in blastocysts by histones after initiation of mTOR signaling, could preserve this activity for activation and concentration of tryptophan into trophoblast cells at the time of trophoblast penetration of the uterine epithelium.

## Conclusions

We propose here that extracellular histones have at least two somewhat surprising functions during early development of blastocysts and their implantation in the uterus. First, free histones protect blastocysts and the uterus from the adverse effects of unwanted inflammation caused by infection. Histones appear in abundance in secretions of the uterus when it is receptive to blastocyst implantation. Thus, these macromolecules provide protection from infection when it is needed for peri-implantation development to continue. Second, histone secretion by the uterus beginning about 15 h before blastocyst implantation could cause the observed suppression of amino acid transport system B^0, +^ activity in blastocysts *in utero*. Removal of histones from the implantation chamber at the time motile trophoblasts penetrate the uterine epithelium would reactivate system B^0, +^ to take up tryptophan. Tryptophan is needed for T-cell proliferation, so its uptake and metabolism by blastocysts would help to prevent their immunologic rejection when they are most vulnerable owing to trophoblast penetration of the uterine epithelium. Because of these possible beneficial actions of extracellular histones, efforts to mitigate histone cytotoxicity in the reproductive tract should be cautious.

## Author contributions

The author confirms being the sole contributor of this work and approved it for publication.

### Conflict of interest statement

The author declares that the research was conducted in the absence of any commercial or financial relationships that could be construed as a potential conflict of interest.
